# Is there a relationship between psychological factors and TMD?

**DOI:** 10.1002/brb3.1360

**Published:** 2019-07-24

**Authors:** Anna Sójka, Bogusław Stelcer, Marco Roy, Ewa Mojs, Mariusz Pryliński

**Affiliations:** ^1^ Department of Prosthodontics Poznań University of Medical Sciences Poznan Poland; ^2^ Department of Clinical Psychology Poznań University of Medical Sciences Poznan Poland

**Keywords:** 4DSQ, sense of coherence, stress, student population, temporomandibular disorders

## Abstract

**Introduction:**

Medical students are in a very demanding environment and are affected by high degree of stress. High levels of anxiety can affect a student's academic performance and also increase the risk of other health‐related problems. This study aims to evaluate, thanks to the intensity of stress manifestations (4DSQ) together with the sense of coherence (SOC), the prevalence of TMD and oral parafunctions in students enrolled in the University of Medical Sciences. Moreover, it aims to evaluate the relationship between the psychosocial manifestations of stress and sense of coherence in relation to gender.

**Materials and Methods:**

A total of 324 students of Poznań University of Medical Sciences participated in this study. Students were assessed using a three‐part questionnaire: one was to assess symptoms of TMD, the second part was 4DSQ, and the third was a SOC Questionnaire.

**Results:**

About one‐third of the students in this study presented symptoms of TMD and perceived more intensively symptoms of distress, anxiety, somatization, and depression. They presented a higher level of somatic symptoms and a lower level of Sense of Coherence than students without TMD symptoms.

**Conclusion:**

There is a strong negative relationship between the sense of coherence and the level of perceived distress, anxiety, somatization, and depression. Female students attending Medical School showed a higher level of somatization of stress but with a higher capacity to overcome challenges as compared to men.

## Graphical abstract

We studied the intensity of stress manifestations (4DSQ) together with the sense of coherence (SOC), the prevalence of TMD and oral parafunctions in students enrolled in the University of Medical Sciences. A negative relationship between the sense of coherence and the level of perceived distress, anxiety, somatization, and depression. Female students had a higher level of somatization of stress but with an higher capacity to overcome challenges as compared to men.

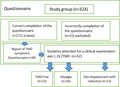

## 1 INTRODUCTION

The term temporomandibular disorder (TMD) is defined as a term that includes a number of clinical problems involving the masticatory musculature, the temporomandibular joint (TMJ) and its associated structures (Durham, 2008; LeResche, 1997). TMD is usually described as a subclass of musculoskeletal disorders, which cause nondental pain in the orofacial region and are included in a newly recommended Diagnostic Criteria for Temporomandibular Disorders (DC/TMD) (Schiffman et al., 2014).

Co‐morbidity factors associated with TMD are as follows: psychological (stress, anxiety, tension), structural (occlusion), repetitive microtrauma from parafunctional habits (both bite and nonbite related), and external traumas (Manfredino, Bandethini, & Cantini, 2009; Manfredini & Lobbezoo, 2009; Molina, Peixoto, & Manzutti Eid, 2011). Several studies of general pain, as well as TMD, have indicated an association with psychosomatic and/or psychosocial aspects (De Leeuw et al., 1994; Dworkin, 2010; Ivkovic, Mladenovic, Petkovic, & Stojic, 2008; Suvinen, Reade, Sunden, Gerschman, & Koukounas, 1997a, 1997b). It is assumed that stress can cause general health complaints such as a headache (Nash & Thebarge, 2006), abdominal pains, and anxiety (White & Farrell 2006). Unpredictable or incomprehensible life situations are also important sources of stress (Hurst, Baranik, & Daniel, 2013; Michie, 2002). Psychological health complaints are highly prevalent, both in the community and in the general medical settings (Heinen, Bullinger, & Kocalevent, 2017). Medical Universities as well are considered stress‐ridden environments (Dahlin, Joneborg, & Runeson, 2005). Terluin identified four symptom dimensions that prove to be sufficient in describing the whole range of common psychological and somatic complaints: “distress,” “depression,” “anxiety,” and “somatization” (Terluin, 1994). The “distress” dimension represents symptoms that result from the strain that is elicited by a stressor, as well as from the psychological effort that has to be put into dealing with that stressor and maintaining an acceptable level of psychosocial functioning (Terluin, 1998). It is well known that medical students often face many difficulties during their medical curriculum (Castadelli‐Maia et al., 2012; Dahlin et al., 2005). If these difficulties are ignored and not dealt with, they are likely to produce further stress that could affect academic activity in individuals who are already in very high‐pressure settings (Dyrbye, Thomas, & Shanafelt, 2006). Research suggests that negative affectivity (the tendency to experience a wide range of negative emotions), may account for a large proportion of the shared variance between self‐perceived stress and physical health reports (Inwood & Ferrari, 2018).

High levels of anxiety can affect a student's academic performance and also increase the risk of other health‐related problems. It is observed that the increase in the incidence of chronic orofacial pain in temporomandibular disorders (TMDs) has an impact on the quality of life and on the general health of subjects. It is observed that the most commonly reported response to a stressful event were intrusive and avoidant thoughts. Intrusion includes unbidden thoughts and dreams (Fong & Loi, 2016; Heinen et al., 2017). Stress, somatic distress, and depression may be potential etiological risk factors for TMDs‐related pain. Psychological factors are more obvious and prominent in patients experiencing TMD chronic pain (Zakrzewska, 2002).

On the other hand, there is a general discussion going on about health resources among student populations. One of the most prominent conceptualizations was a theory developed by Aaron Antonovsky who tried to find a solution as to why some people, regardless of major stressful situations and severe hardships, stay healthy, while others do not. He introduced the concept of Sense of Coherence—measured by the Orientation to Life Questionnaire (SOC) (Antonovsky, 1979, 1987). SOC is determined from the so‐called Salutogenetic approach, which is the search for sources of health, rather than causes for diseases. SOC consists of three core components: (a) comprehensibility, which is a person's perception that the internal or external environment is structured, predictable, and consistent; (b) manageability which is the belief that resources are available for dealing with problems; (c) meaningfulness which is a person's perception that life's events have meaning and are worth investing energy in (Antonovsky, 1979, 1987).

The aim of this study was to evaluate the prevalence of TMD symptoms and oral parafunctions in students enrolled in the University of Medical Sciences and its relationship with the intensity of stress manifestations (4DSQ) together with the sense of coherence (SOC). It also aimed to evaluate the relationship between the psychosocial manifestations of stress and sense of coherence in relation to gender.

The null hypothesis is that there is a correlation between psychological factors and TMD and that there is no relationship regarding sense of coherence and TMD in the research population.

## 2 MATERIALS AND METHODS

### 2.1 Participants

A total of three hundred and twenty‐four students of Poznań University of Medical Sciences participated in this study. A survey for research was given to students during a periodic analysis on the state of their health at the Occupational Medicine Clinic which operates at the Medical University in Poznań. Filling out the questionnaire was both voluntary and anonymous; survey questions did not include the personal realm. Students were assessed using a three‐part questionnaire: one was to assess symptoms of TMD, the second part is 4DSQ, and the third is a SOC Questionnaire (Antonovsky, 1987; Czachowski, Izdebski, Terluin, & Izdebski, 2013; Terluin, 1996, 1998). After verifying that the symptoms of TMD as reported in the questionnaire were present, a dentist carried out a clinical examination that included palpation of the masticatory muscles and the TMJs. These examinations were performed by a dentist trained in the DC/TMD axis I protocol (Schiffman et al., 2014).

Ethical considerations are in agreement with the Helsinki Declaration. Approval was received from the Bioethical Committee of the Poznan University of Medical Sciences, as to include studies on healthy people (859/15, 01.10.2015). The anonymity of the participants was preserved and no financial nor any other burden was placed upon them.

To prevent the respondents from giving an induced answer no time limit was set in filling out the questionnaire. Participants were informed about the objectives of the study and about how to participate, those who agreed signed a consent form, which was attached to the questionnaire. To each participant was given a code number, in order to safeguard his/her identity. Exclusion criteria were a history of serious neurological disorders; systemic disorders; cervical spine injuries or postural deformities; the presence of drug abuse; or of a comorbid condition such as malignant disease; and pregnancy or nursing.

### 2.2 Research tools and questionnaire survey

The first part of the questionnaire was focused on the etiological factors and symptoms of temporomandibular disorders (TMD) according to axis II of (DC/TMD), and included occurrence of bite and nonbite parafunctions and the presence of voltage stress (Schiffman et al., 2014). The questionnaire also collected information, such as the status of the patient's general health and previously received treatment. Parafunctional habits were categorized according to the following classification: bite habits (that involve the contact of opposing teeth), such as clenching or grinding the teeth when asleep or/and habitual teeth clenching, pressing, touching, or holding the teeth together other than while eating; nonbite habits (which do not involve opposing teeth contact) such as tongue, lip/cheek biting, biting on objects, nail‐biting. Students were asked to check the items that were most relevant to their conditions.

#### 2.2.1 Examination of TMD

An Examination Form of Diagnostic Criteria for Temporomandibular disorders axis I (DC/TMD) was used in order to assess the stomatognathic system clinically. The clinical examination required about 10–15 min. The classification included two major groups: 1. muscle disorders, 2. disc displacements. Digital palpation of the TMJ was done with the middle and index fingers, while listening for audible sounds during opening and closing of the mouth (acoustic symptoms of TMD). Since the TMD pain algorithm requires that any pain provoked with palpation or mouth opening must be a familiar pain (i.e., pain similar or like the patient's pain complaint), the patients were asked whether they experienced any pain during the clinical tests and if so, if this was the pain for which they sought help (Schiffman et al., 2014). To measure maximum mouth opening (without overbite), each subject was asked to open the mouth as wide as possible while the examiner measured the maximum distance from incisal edge of the maxillary central incisors to the incisal edge of the mandibular central incisors at the midline. A disposable scale was used to obtain this measurement (Manfredini, Borella, Favero, Ferronato, & Guarda‐Nar‐dini, 2010; Manfredini, Winocur, Ahlberg, Guarda‐Nardini, & Lobbezoo, 2010). Opening deviation (movements symptoms of TMD) was defined as the displacement of the mandible of at least 2 mm to the right or left of an imaginary vertical line when the mandible reached half of its vertical opening.

#### 2.2.2 Four‐Dimensional Symptom Questionnaire

The second tool we used to assess the prevalence of stress and its psychosocial manifestations was the questionnaire Four‐Dimensional Questionnaire (4DSQ). In general practice, the 4DSQ enables the physician to distinguish between psychiatric illness and uncomplicated stress‐related disorders (Terluin, 1996, 1998). This tool contains 4 scales: stress, depression, anxiety, and somatization. The 4DSQ is a self‐report questionnaire comprising 50 items distributed over four dimensions: a distress scale (16 items), a depression scale (6 items), an anxiety scale (12 items), and a somatization scale (16 items; Czachowski et al., 2013; Terluin, 1996). Items are worded as questions about specific symptoms similar to those asked in everyday primary care practice. Items were scored on a 5‐point Likert type scale (0 = “No,” 1 = “Sometimes,” 2 = “Regularly,” 3 = “Often,” 4 = “Very often or constantly”). The reference period was “the past week”. To reduce the possible influence of exaggerating response tendencies on summed scores, all item scores of “3” and “4” are recorded into a score of “2” before calculating summed scores per dimension. Because each item provides 0, 1, or 2 points to the scale summed score, the score range of each scale is twice the number of items in that scale. For example, the score on the 16‐item distress scale ranges from 0 to 32. The 4DSQ has proper psychometric qualities, it is a reliable and internally consistent self‐report questionnaire (Czachowski et al., 2013; Terluin, 1996, 1998).

#### 2.2.3 Sense of coherence orientation to life questionnaire

In order to assess the individual health resources of participants and the sense of coherence in the challenges posed by being in our research group during the study of medicine, we used the 13‐item Orientation to Life Questionnaire which measures Sense of Coherence (SOC) in its short version proposed by Aaron Antonovsky. The sum of all items provides a score ranging from 13 to 91, higher scores indicating a stronger SOC (Antonovsky, 1979, 1987, 1993).

The presence of parafunctions, the occurrence of etiological factors of TMD, the prevalence of TMD according to DC/TMD, the prevalence of a headache, stress, amount of stress in each TMD elicited from history and clinical examinations are presented in the tables as counts (in absolute numbers) while frequencies are expressed in percentages. The aim was to analyze the relationship between the resources of coping with stress (manageability, comprehensibility, and meaningfulness) and its manifestations, that is, stress, depression, anxiety, and somatization. Due to the lack of normal distribution of these variables, the Spearman rho correlation coefficient was used. The Mann–Whitney test was used to analyze the differentiation of the level of psychosocial manifestations of stress and the resources available to cope with it on the basis of being male or female because it was found that the variables tested were not compatible with the normal distribution. The data obtained from the study were then analyzed using Statistica (data analysis software system), v.10.0, StatSoft (2011). The level of significance was set at *p* ≤ .05. Statistically significant differences between males and females in regards to symptoms of the temporomandibular disorder and parafunctional habits were determined by chi‐squared test with a *p*‐value < .05.

## 3 RESULTS

### 3.1 Clinical descriptive analyses

**Figure 1:**
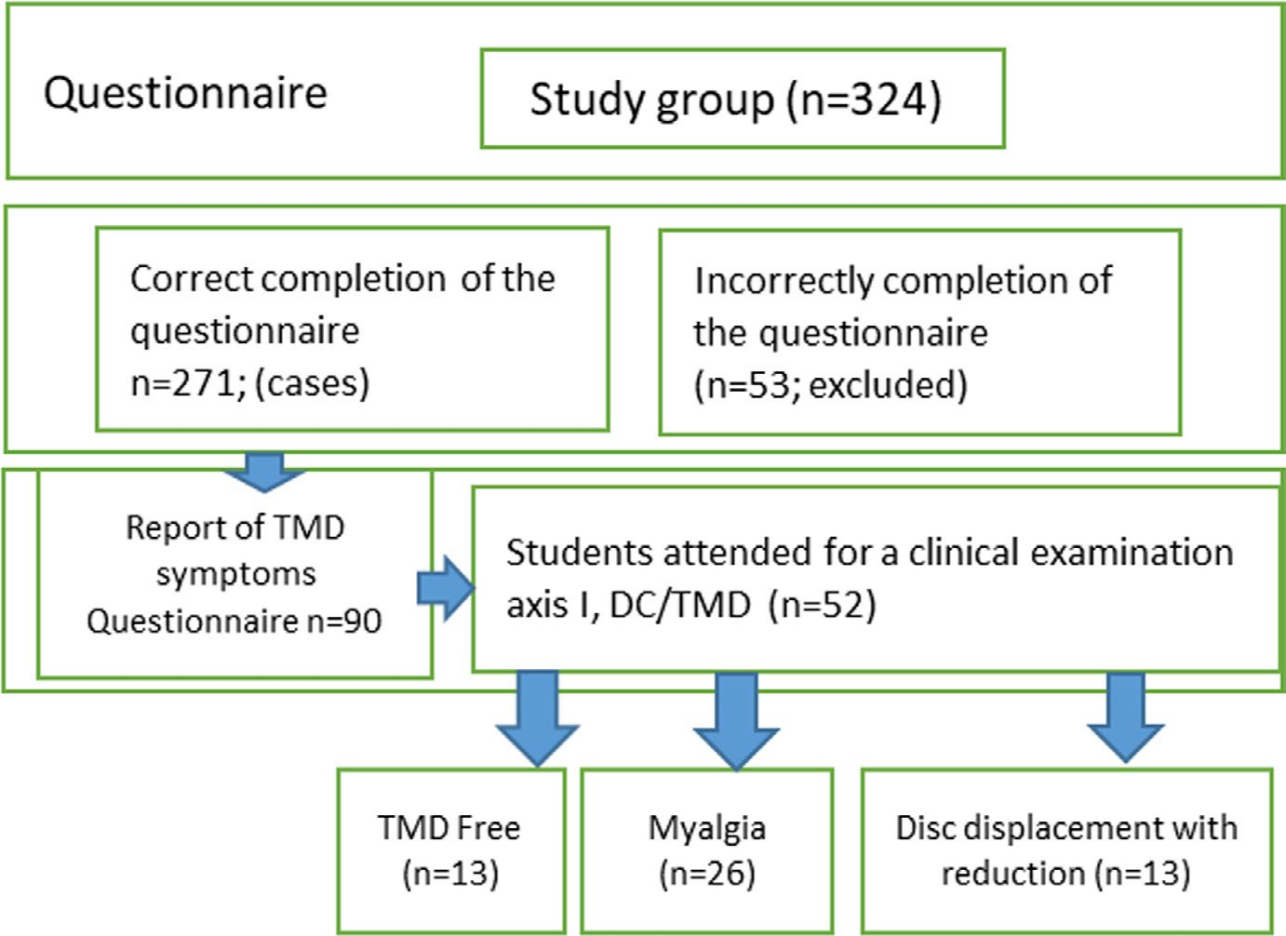
Flow chart of the study participants and inclusion in regards to symptoms of temporomandibular disorder according to axis I of DC/TMD classification

A total of 324 students participated in this study. The study sample consisted of 247 (76.2%) students studying Medicine and 77 (28.1%) students from other Medical related courses, at Poznań University of Medical Sciences (e.g., nursing, public health, physiotherapy, dentistry) Fifty‐three (16.4%) of the participants were excluded due to incorrect compilation of the questionnaire. The final study group consisted of 271 students. There were 180 female and 91 male students and their age ranged between 18 and 32 (mean = 21.28) years old. Based on the questionnaire, symptoms of TMD were observed in 90 students and they were referred for clinical evaluation. Only 52 students attended clinical examination. The diagnosis of Myalgia (M) was clinically established in 26 participants, Disc Displacement with Reduction (DDR) was diagnosed in 13 students, and 13 subclinical TMD were participants who reported symptoms but who were negative for TMD when examined (Figure 1).

There was no statistically significant difference between the number of factors of TMD between females and males. Moreover, males presented more frequently bite parafunctions (27.5%) as compared to females (12.8%). However, males presented nonbite parafunctions of (45.1%) while females scored higher (55%). The amount of stress in both groups was similar, in females being 91.1% and 81.2% in males (Table 1). Symptoms of TMD such as the amount of stress and nonbite parafunctions were more intense in females than males. These differences were not statistically significant. However, there was a significant difference (*p* = .043) between the occurrence of headaches between males and females (Table 2).

**Table 1:** Prevalence of factors of temporomandibular disorder according to questionnaire compared between men and women

Factors of TMD	Women (*N* = 180) (%)	Men (*N* = 91) (%)	*p*‐Value
Trauma	2 (1.1)	5 (5.5)	.081
Amount of stress	166 (92.2)	74 (81.3)	.493
Bite parafunctions	23 (12.8)	25 (27.5)	.140
Nonbite parafunctions	99 (55.0)	41 (45.1)	.156

Note

*p*‐Value < .05.

**Table 2:** Prevalence of symptoms of temporomandibular disorders according to questionnaire comparing men and women

Symptoms of TMD	Women (*N* = 180) (%)	Men (*N* = 91) (%)	*p*‐Value
Pain symptoms	35 (19.4)	13 (14.3)	.378
Pain symptoms after night	11 (6.1)	3 (3.3)	.485
Acoustic symptoms	32 (17.8)	8 (8.8)	.074
Movement symptoms	17 (9.4)	3 (3.3)	.114
Headache	84 (46.7)	30 (33.0)	.043*

Note

*p*‐Value < .05.

* Significant result.

The prevalence of chronic pain located in the facial area (Chronic Pain Grade) according to DC/TMD axis II indicates dysfunction. The interpretation of data, the four grade scale of chronic pain used in DC/TMD questionnaire was 0—for the absence of pain, 1—low‐intensity pain, 2—high‐intensity pain. In the presence of a muscle disorder like Myalgia (M), chronic low‐intensity pain was diagnosed in 73.0% of the subjects while high‐intensity pain was found in 23.1% of the subjects. Among students presenting disc displacement with reduction (DDR), low‐intensity pain was diagnosed in 46.2% and with high‐intensity pain present in 61.5% of the subjects. In the presence of DDR, the high‐intensity pain was highly present but without significant difference. There was a significant difference (*p* < .001) between the number of acoustic symptoms of TMD present in subjects with Myalgia (7.7%) and in all subjects, it being (100%) with DDR. Moreover, a significant difference was found (*p* = .027) in movement‐related symptoms of TMD between subjects with Myalgia and DDR (Table 3).

**Table 3:** Prevalence of symptoms of TMD in each TMJ disorder according to axis I DC/TMD

TMD diagnoses (axis I DC/TMD)	Pain symptoms of TMD VAS	GCPS: Graded Chronic Pain Scale: 1,2	Acoustic Symptoms of TMD *N *(%)	Movement symptoms of TMD *N* (%)	Headache *N* (%)	Parafunctions *N* (%)	Amount of Stress
Myalgia (M) (*n* = 26)	3.35 (±1.18)	1:19 (73.0%) 2:6 (23.1%)	2 (7.7%)	3 (11.5%)	17 (65.4%)	19 (73.0%)	4.4 (±6.6)
Disc displacement with reduction (DDR) *N* = 13	3.38 (±1.19)	1:6 (46.2%) 2:8 (61.5%)	13 (100.0%)	9 (69.2%)	7 (53.8%)	9 (69.2%)	5.5 (±1.9)
Statistic	*t*(44) = 0.082	*χ* ^2^(1) = 2.96	*χ* ^2^(1) = 11.42	*χ* ^2^(1) = 4.91	*χ* ^2^(1) = 0.6387	*χ* ^2^(1) = 0.063	*t*(44) = 1.89
*p*‐Value	.935	.085	<.001**	.027*	.424	1	.065

Note

*p*‐Value < .05.

^*,**^Significant result.

In the second part of our study, there is the comparison between the intensity of stress and its psychosomatic symptomatology among students in the group with TMD symptoms (N = 90) and without TMD symptoms (*N* = 181). A question was raised as to whether students reporting TMD symptoms differ in terms of how they answer questions from the 4DSQ questionnaire and on the Life Orientation Questionnaire (sense of coherence). The results confirmed the theoretical assumption that a stronger level of distress and a lower sense of coherence will be present in the group of students with TMD symptoms. Table 4 contains the values of chi‐square statistics confirming the existence of a relationship between variables. This allows us to look at students with symptoms of TMD as those who probably experience stress in their daily lives and which the consequences of which they cannot cope with. In addition, their sense of coherence is significantly lower.

**Table 4:** Psychosocial manifestations of stress in relations to sense of coherence in the groups with and without symptoms of TMD

4DSQ	Chi‐square	*df*	Significance asymptotic
Stress	14.34	1	.00
Depression	14.60	1	.00
Anxiety	12.87	1	.00
Somatization	37.15	1	.00
Meaningfulness	6.23	1	.013
Comprehensibility	11.84	1	.001
Manageability	5.99	1	.014

Note

*p*‐Value < .05.

Abbreviation:* df*, degrees of freedom.

Results show that there are differences between both groups on each dimension of the 4DSQ questionnaire and Orientation to Life Questionnaire. Students with TMD presented a statistically significant different way of responding to the questionnaire items in comparison with students who did not suffer from TMD symptoms.

#### 3.1.1 Intensiveness of somatic symptoms

Similar relationships were confirmed in relation to the whole measured student population. Also checked was which relationship occurs among the examined population of students and the factors contributing to the sense of coherence (manageability, comprehensibility, meaningfulness) as well as to the manifestations of stress, that is, stress tension, depression, anxiety, and somatization. Due to the lack of normal distribution of these variables, the Spearman rho correlation coefficient was used. The results show that there are statistically significant relationships between all variables associated with the units of Orientation to Life Questionnaire (health resources) and the intensity of stress, depression, anxiety, and somatization measured by the 4DSQ questionnaire. With the increase in the sense of coherence, manageability, comprehensibility, and meaningfulness, there is a reduction in the level of stress, depression, anxiety, and somatization in the measured subjects Table 5.

**Table 5:** Psychosocial manifestations of stress in relation to sense of coherence

4DSQ	Stress	Depression	Anxiety	Somatization
Meaningfulness	−.386*	−.347*	−.301*	−.266*
Comprehensibility	−.413*	−.375*	−.389*	−.367*
Manageability	−.390*	−.329*	−.290*	−.322*

* Variable statistically important on level *p* < .01; *n* = 271.

In the next step, we analyzed the psychosocial manifestations of stress in relation to the sense of coherence between students divided into two groups depending on the level of TMD, namely Myalgia and DDR. Somatization was statistically significant (*p* = .044) between these 2 groups. Students from the DDR group had a slightly higher tendency to somaticize than students from the Myalgia group (Table 6).

**Table 6:** Prevalence of psychological factors in each TMJ disorder according to DC/TMD axis I diagnostic criteria

	Myalgia (*n* = 26)	Disc displacement with reduction (DDR) (*n* = 13)	*p*
	M [*SD*]	M [*SD*]	
Stress	2.0 [0;26]	1.0 [0;8]	.168
Depression	0.0 [0;1]	0.0 [0;1]	.784
Anxiety	0.0 [0;1]	1.0 [0;1]	.192
Somatic symptoms	1.5 [0;3]	4.0 [2;5]	.044*
Meaningfulness	23.0 [11;28]	25.0 [19;28]	.567
Comprehensibility	26.5 [16;34]	28.0 [22;33]	.813
Manageability	22.0 [9;28]	24.5 [19;27]	.742

Note

*p* < .05.

* Significant result.

### 3.2 Psychosocial manifestations of stress and sense of coherence dependent on gender

In the next step, the hypothesis about the differentiation of the level of psychosocial manifestations of stress in relation to the sense of coherence due to gender was verified. The analysis was carried out on 271 students. For the analysis, the Mann–Whitney test was used because there was a lack of general agreement on the normal distribution of the variables tested. The obtained results entail the conclusion that men and women differ significantly in terms of the level of meaningfulness and somatization. In females, the level of these traits was significantly higher than in males (see Table 5). For the other variables, no significant gender differences were found.

## 4 DISCUSSION

We analyzed the data employing various techniques including frequency distributions, means, ranges, standard deviations (SDs), and significance tests such as chi‐square and analysis of variance. In addition, a hypothesized model was utilized as a statistical method to determine the underlying relationships among the variables. In keeping with customary usage, for the statistical analysis, the level of significance was established at *p* ≤ .05.

### 4.1 TMD

Literature supports the notion that psychological stress negatively impacts physical health (de Ridder, Geenen, Kuijer, & van Middendorp, 2008). A widely used definition of stressful situations is when the demands of the situation threaten to exceed the resources of the individual. All people are exposed to stressful situations at the social, community, and interpersonal level. Health professionals have high levels of anxiety, which can start during University years and bring repercussions not only on academic performance but also in increased risk for other diseases (Dyrbye et al., 2006; Sherina, Rampa, & Kaneson, 2004). Our results confirm the data obtained in previous studies showing the presence of stress among medical students. We presented the comparison between students presenting symptoms of TMD (*N* = 90) and those not representing symptoms (*N* = 181). According to our theoretical predictions, it was confirmed that the sense of coherence constitutes a health resource for the surveyed students. Students without symptoms of TMD achieved higher scores on the Antonovsky Life Orientation Questionnaire scale. Amongst students with TMD, stress, anxiety, depression, and somatization were relevantly statistically higher (Table 5). This research confirmed the negative effect of the relationship between stress and health resources. This finding is comparable to the results found by Bodyk‐Cupak et al. in nursing students, where a low level of self‐perceived stress had a significantly higher level sense of self‐efficacy (Bodyk‐Cupak, Majda, Zalewska‐Puchała, & Kamińska, 2016). Temporomandibular disorders (TMD) are associated with a multifactorial etiology and with pathophysiological, social, cultural, and psychological components (Barbosa, Miyakoda, Pocztaruk, Rocha, & Gavião, 2008). Psychological factors in TMD situations may be divided into behavioral symptoms, such as bruxism, emotional, such as stress, anxiety and depression, and cognitive, with memory‐related aspects. Psychological studies have shown that patients with TMD have similar psychological profiles and psychological dysfunction as other chronic musculoskeletal pain disorders (Manfredini & Lobbezoo, 2009). The increased incidence of pain associated with TMD has a major impact on the quality of life of the population (Durham, 2008; Kafas, Kafas, Christofides, Chiotaki, & Theodoridis, 2008). This aspect was not the focus of this study but the impact of stress on a student's quality of life is worth to be considered for a future research. Our results seem to confirm the dependence indicated in the study Terluin et al. (Terluin, Brouwers, van Marwijk, Verhaak, & van der Horst, 2009; Terluin, Van Rhenen, Schaufeli, & De Haan, 2004) in which the effects of stress can be explained by examining the four dimensions of psychological response: distress, depression, anxiety, and somatization. The relationship between TMD and its symptoms (measured by the 4DSQ questionnaire) were confirmed by the somatic symptoms found in students experiencing stress. Similar results were presented in literature (Giannakopoulos, Keller, Rammelsberg, Kronmüller, & Schmitter, 2010), in which is described the relationship between perceived pain and depression. In our study, female students were more intensely affected by somatic symptoms and headaches (Tables 2 and 7). In terms of a sense of coherence, it was found that female students have their main source of health (SOC) in their sense of meaningfulness. This is in contrast with the study by Anderson‐Darling et.al. (Anderson‐Darling, McWey, Howard, & Olmstead, 2007) where social relationship, quality of friendship and quality of relationship with parents played the main role. Instead, the group of students in our research resulted autonomous.

**Table 7:** Results of the rating scale of the Sense of Coherence Scale. Descriptive statistics by gender

4DSQ/SOC	Women (*N* = 180)	Men (*N* = 91)	*p*‐Value
	*M* (*SD*)	*M* (*SD*)	
Amount of stress	4.9 (±2.2)	4.3 (±2.7)	.387
Depression	0.3 (±0.9)	0.3 (±1.0)	.805
Anxiety	0.7 (±1.9)	0.5 (±1.3)	.173
Somatic symptoms	3.0 (±3.3)	2.1 (±2.3)	.009**
Meaningfulness	24.0 (±3.1)	22.6 (±4.0)	.005**
Comprehensibility	27.1 (±5.0)	26.5 (±5.4)	.405
Manageability	22.5 ± 3.7	21.7 ± 4.6	.139

Note

*p* < .05.

** Significant result.

Bruxism is a frequent factor found in TMDs; it refers to a nonfunctional grinding and clenching of the teeth. Individuals usually clench their teeth when they are sleeping, but this can occur at some stage in early awakening (Lobbezoo et al., 2013). Symptoms are severe on awakening and over time, the continuous pressure can damage the TMJs (Marbach, Raphael, Janal, & Hirschkorn‐Roth, 2003). Nonbite parafunctional habits are comprised of biting foreign objects, pressing the tongue against the teeth and lip biting (Motta et al., 2013). In our study, 62.0% of students were found to have a bite and nonbite parafunctions. Bite parafunctional habits (bruxism) were present in 17.7% of cases while 51.7% of subjects revealed nonbite habits. Occlusal parafunctions appeared more frequent in male students, whereas nonbite parafunctions are more frequent in female students. No significant difference was noted in the prevalence of bite and nonbite parafunctional habits between female and male students. A study by Manfredini, Cantini, and Romagnoli (2003) found that bruxism has a well‐built relationship with muscle disorders and with disc displacement and joint pathologies. In our study, prevalence of parafunctions was similar in both groups, being 73% in myalgia group and 69.2% in DDR group. In literature, the prevalence of TMD in females is reported to be almost twice as that of males (Graue, Jokstad, Assmus, & Skeie, 2016; Huang, LeResche, Critchlow, Martin, & Drangsholt, 2002; Sanders & Slade, 2011). This high prevalence among females is hypothesized to be due to the physiologic uniqueness of each individual such as their regular hormonal variations, different characteristics of connective tissue, and muscular structure (Magnusson, Egermark, & Carlsson, 2000; Pedroni, De Oliveira, & Guaratini, 2003). In our studies, the frequency of TMD pain, movement, and acoustic symptoms in female compared with male students was higher but the difference was not statistically significant. Among students presenting disc displacement with reduction (DDR), the high‐intensity pain was in 61.5% of the subjects. Students with DDR reported a higher intensity of pain when compared to students with myalgia; however, it was not significant. Instead there was a significant difference between the number of acoustic symptoms of TMD between students affected with Myalgia when compared to those with DDR. Several studies have shown that TMD patients with high levels of pain‐related disability measured by Graded Chronic Pain Scale (GCPS) show a high level of depression, somatization, sleep dysfunction, worry, and catastrophizing thoughts (Kotiranta et al., 2015; Manfredini, Winocur, et al., 2010). On the other hand, Manfredini et al. have shown that the GCPS was not significantly associated with depression in TMD patients, and the author attributed the controversy of the association between depression and GCPS to the small sample size (Manfredini, Borella, et al., 2010). A study by List, Wahlund, and Larsson (2001) found that adolescents with TMD reported significantly higher levels of stress and psychosocial problems. Our results confirmed this hypothesis. Students with diagnosed DC/TMD presented significantly higher symptoms of stress and lower health resources described as the sense of coherence (Tables 4 and 5).

It would seem necessary to organize a method of health prophylaxis for students, especially for those who study high demanding disciplines. Nilsson et al. indicated that depressive symptoms and somatic complaints co‐occurred in girls more than boys with TMD pain (Nilsson, Drangsholt, & List, 2009). Some research indicates that psychosocial factors play a key role in TMD, especially when it becomes more chronic in nature. Additionally, it is suggested that psychosocial factors can intensify both pain behavior and the amount of pain felt (Leary & Hoyle, 2013; Rollman & Gillespie, 2000). Differences in reaction to stress depend on heredity, sex, and social‐natural environment. Hence, the degree of contribution of different factors to TMD may be related to individual differences among people. The differences that exist between males and females in the perception, expression, and tolerance of pain are likely influenced by a variety of social and psychological processes (Miller & Newton, 2006). Gender roles have also been associated with a pain response, with the masculine gender norm dictating increased tolerance for pain among males, whereas feminine gender norms accept pain as a normal part of life and are more permissive of pain expression (Myers, Riley, & Robinson, 2003). Our studies have shown that women are likely more prone to express somatic symptoms than men. These differences are worth to be explored in future studies.

### 4.2 Stress and sense of coherence

The salutogenic stress model marks a departure from stressor‐focused research. This model emphasizes health promoting and individual strengths that abate the harmful effects of stress. Salutary factors include a coping style, sense of coherence, and social support. Coping styles with stress are defined as cognitive and behavioral efforts to recover homeostasis between demands and the resources of an individual in a situation, he or she perceives as being burdensome and exceeding with regard to their current available resources. The concept of sense of coherence is focused on a different perspective; it is rather a concept describing health resources of the subject in her/his confrontation with stress (Antonovsky, 1979, 1987, 1998). The adoption of a particular coping style is an attempt to eliminate or reduce them. According to Kindler et al., diagnosis, prevention, and treatment of TMD pain must take into consideration depression and anxiety symptoms (Kindler et al., 2012). Our results show that there are statistically significant relationships between manageability, comprehensibility, and meaningfulness associated with the student's resources to cope with stress and the intensity of stress, depression, anxiety, and somatization measured by the 4DSQ questionnaire. Higher sense of coherence was correlated with a lower manifestation of somatic symptoms of stress.

Most previous studies report stronger Sense of Coherence (SOC) among men than women (Eriksson, 2007), although in some the association between SOC and health‐related factors is stronger among women (Kivimäki, Feldt, Vahtera, & Nurmi, 2000). There has also been speculation that in terms of health, SOC has more relevance to women than to men. Antonovsky did not explicitly analyze the meaning of resistance resources in terms of gender (Antonovsky, 1987). Our research gives evidence that women studying at Medical University have a higher level of sense of coherence than the research population of male students. The study showed that female medical students had a significantly higher level of the sense of coherence, in specific the sense of meaningfulness (*p* = .005). This means that for these women it is more important to put effort and commitment to meet the demands of life. These results confirmed the findings of previous studies (Marcinkowska, Lau, & Jośko‐Ochojska, 2013). Women are more determined in undertaking challenges, and they therefore put more effort into solving the problems or in dealing with them. Because this component of the sense of coherence is described as having motivational and emotional aspects of coping. It seems reasonable to conclude that female students at Poznań University of Medical Sciences do so with greater motivation and they are more prone to overcome the emerging difficulties. Moreover, female students present themselves very determined and are more prone to successfully complete their studies; however they reported a higher incidence of somatic symptoms than men (*p* = .009). This study has confirmed the fact that students of medical faculties experience stress, anxiety, negative emotions, and symptoms of depression (Anderson‐Darling et al., 2007; Humphris et al., 2002). Our findings support the opinion that the entity of this problem needs to be properly recognized in the face of emerging evidence of the growing problem of stress and psychosomatic dysfunctions in medical students and other fields of study (Ofili, Oriaifo, Okungbowa, & Eze, 2009). In our study, the sense of coherence may be related to different ways of coping with tension and stress between males and females.

### 4.3 Psychosomatic symptoms and sense of coherence

Sense of coherence was used to signify the degree of organization or disorganization and health resources in the lives of male and female university students. Global SOC, meaning comprehensibility, manageability, and meaningfulness were slightly higher among females students but without any significant difference. Moreover, level of anxiety and somatization of the symptoms was higher in female students. However, they experienced a lower amount of stress and depressive moods in comparison with male students. The statistical analysis indicated that there was a negative relationship between all variables describing psychosocial manifestations of stress measured by 4DSQ and SOC. It confirms that high level of sense of coherence is a protecting factor from stress and risk of somatization. Findings give evidence that the sense of coherence negatively correlates with physical and emotional symptoms. Students presenting symptoms of TMD reported a lower level of sense of coherence. In our research, it was statistically significant that female students expressed a higher level of somatic symptoms than men and a higher level of meaningfulness. Our study shows inverse correlation between somatization and sense of coherence among students with TMD. Similar findings have been presented in the study of Su et al., where somatization and depression were associated with pain intensity and pain‐related disability (Su, Lobbezoo, Vanwijk, Vanderheide, & Visscher, 2017).

In clinical practice, early behavioral intervention is thought to reduce the risk of patients developing persistent or chronic pain (Hasenbring, Hallner, & Klasen, 2001). A psychosocial disorder should be regarded as a very important comorbid condition contributing to TMD onset. Nevertheless, clinicians should promptly assess the somatization or depression conditions of patients with high pain intensity or pain‐related disability to provide them with a multiple approach therapy including both psychological support and physical treatment (Fillingim et al., 2013). Findings of Bortsov et al. suggest that subjects with prevailing sympathetic tone are more likely to express more somatic symptoms, namely higher pain intensity or unpleasantness, poor sleep quality; concluding that further studies should focus on whether an individual autonomic profile could be a prognostic factor for TMD progression and treatment outcomes (Bortsov et al., 2017). Study of Gil‐Martínez provides important information regarding psychosocial factors that appear in patients with chronic TMD, which could be disability predictors (Gil‐Martínez, Grande‐Alonso, López‐de‐Uralde‐Villanueva, López‐López, & Fernández‐Carnero, 2016). The author suggests that these factors should be considered as likely predictors in the evaluation and treatment of these patients.

## 5 LIMITATIONS

Several limitations of this study should be noted. First, the survey done in this research is not able to verify causal relationships between expression of the somatic symptoms and meaningfulness in female students. Therefore, the results should be interpreted with caution. Second, this study was conducted in a population of University students in Poland attending different medical specialties and years. It is unclear whether these results can be generalized in other cultures or to other populations. Further studies are needed to confirm these findings. In the end, the amount of missing data may indicate potential selection bias. And a large representative sample of university students is urgently needed to achieve more conclusive results.

## 6 CONCLUSION

This study provides evidence that medical students' somatic stress and emotional distress levels are generally high and that personal resources and sense of coherence are acting as a buffer. Our results suggest the necessity to provide the students with individual interventions, most of them need support in dealing with the challenges of the medical curriculum as well as addressing structural determinants of stress such as course load and timing of the exams. In fact, there is important a high request to prevent psychosocial and organizational stress in students. The results are in line with the data present in literature. High incidence of TMD symptoms, bite and nonbite parafunctional habits, was found. This paper underscores the need for a multilevel diagnostic and therapeutic approach. It is of great importance a thorough examination of the stomatognathic system and the identification of a destructive influence of parafunctional habits in universities students. DC/TMD classification has proven to be an effective tool in diagnosing TMD.

## CONFLICT OF INTEREST

None declared.

## DATA AVAILABILITY STATEMENT

The data used in the current study available from the corresponding author on reasonable request.
